# iCare® Home vs Goldmann applanation tonometry: Agreement of methods and comparison of inter-observer variation at a tertiary eye centre

**DOI:** 10.1177/11206721221099252

**Published:** 2022-05-03

**Authors:** Laurence Quérat, Enping Chen

**Affiliations:** Division of Eye and Vision; Department of Anterior Segment Disorders, Glaucoma, Neuro-Ophthalmology and Oculoplastics, 97092 Department of Clinical Neuroscience, Karolinska Institute, St Erik Eye Hospital, SE-171 64 Solna, Sweden

**Keywords:** intraocular pressure, GAT, rebound tonometry, self-tonometry, iCare® Home

## Abstract

**Purpose:** To investigate whether the inter-observer variation is similar between the Goldmann applanation tonometer used by healthcare staff and the iCare® Home tonometer used by glaucoma patients, volunteers and healthcare staff. **Methods:** Sixty-one participants were recruited to the study, including 24 glaucoma patients. Seven participants were excluded. For each participant, intraocular pressure (IOP) was measured on the same occasion by two different healthcare staff using GAT as well as by a healthcare staff and the participant using the iCare® Home tonometer. **Results:** Seventy-two per cent of iCare® measurements were within 3 mmHg of the GAT measurements. There was a statistically significant difference between the trainers’ measurements made with iCare® Home and those made with GAT (*p* < 0.001), as well as between the GAT measurements made by trainers and those made by extra personnel (*p* = 0.017). The strongest correlation was between iCare® Home participants’ and trainers’ measurements (0.934). The correlation between different users with GAT was lower (0.769). The inter-user agreement was excellent for iCare® Home users (95% CI 0.93, ranging from 0.880 to 0.959) and moderate for GAT users (95% CI 0.741, ranging from 0.558 to 0.849). **Conclusion:** Our study found that tonometry with iCare® Home has similar or less inter-user variation compared with GAT.

## Introduction

Intraocular pressure (IOP) is the only adjustable risk factor in glaucoma management. Different methods to measure the IOP are available, with Goldmann applanation tonometry (GAT) being the gold standard. GAT requires topical anaesthesia and qualified personnel to obtain measurements, limiting its use to healthcare providers, usually during office hours. Rebound tonometry with iCare® devices is an alternative method that does not require anaesthesia. The iCare® Home tonometer allows patients to measure their IOP outside of healthcare premises (e.g. at home, work, etc.), whenever it is needed. Self-tonometry with iCare® Home has been evaluated in recent years. Several studies have shown good agreement between measurements made by patients or healthcare personnel with iCare® Home and measurements made using GAT.^1–11^ However, there is doubt among healthcare providers regarding the reliability of iCare® Home measurements made by patients.

In fact, it is known that none of the existing methods measure the true IOP. Clinically, GAT is regarded as the gold standard for tonometry, as the method is well established. However, like any other method, GAT has its limitations due for example to the influence of corneal properties (thickness, hysteresis) and tear film.^12–15^ Furthermore, GAT has considerable inter-and intra-observer variation. A study by Berry et al. showed differences of 0.71 and 1.17 mmHg when two technicians measured the IOP of the same patients with different instruments.^16^ Another study by Phelps et al. found differences of 2 mmHg or more in 50% of 420 eyes when the IOP was measured by two technicians and 3 mmHg or more in 30% of the same eyes.^17^ Thorburn obtained differences of 0.50 mmHg when the same technician repeated measurements on the same patients and of 0.70 mmHg when two different technicians performed GAT.^18^

In clinical practice from visit to visit, the IOP is often measured with GAT by different healthcare providers, such as ophthalmic nurses, optometrists or physicians. This practice sometimes shows IOP deviations; although such deviations may be partly explained by inter-observer variation, they may nevertheless influence a clinical decision regarding glaucoma treatment. Indeed, Kawai et al. concluded that the greater the number of clinicians measuring the IOP during follow-up visits, the higher the IOP variability.^19^

As inter-observer variation can exist with both GAT (among healthcare professionals) and iCare® Home (between healthcare professionals and patients), the purpose of this study is to investigate whether the inter-observer variation is similar between these two methods.

## Methods

This prospective study was conducted in accordance with the tenets of the Declaration of Helsinki and was approved by the Ethical Review Board of Stockholm (Nr 2019-06184).

### Participants

All consecutive glaucoma patients who were referred to perform an IOP phasing with iCare® Home between April and September 2020 were invited to participate in this study. The majority of these patients were first-time users. Inclusion criteria were primary open-angle glaucoma (POAG), pseudo-exfoliation glaucoma (PEX) and ocular hypertension (OHT). Furthermore, volunteers with no known eye disease were eligible to participate. Written informed consent was obtained from each participant. Seven participants had tested iCare® Home once a few years previously.

### Device

iCare® Home is a handheld tonometer designed for patients to use at home. Rebound tonometry is based on an estimation of the deceleration of a magnetized probe that bounces back from the cornea. A valid result is derived from six IOP measurements. The instrument automatically excludes the lowest and highest IOP values and calculates the mean of the four remaining IOP values. The device only accepts measurements made within a distance of 4–8 mm from the eye. No anaesthetic is required for the measurements.

Goldmann applanation tonometers (Haag Streit) were used and calibrated once a month at the clinic.

### Procedure

For each participant, a total of six measurements were obtained on a single visit. The participants were first introduced to the self-tonometer, and a single measurement with iCare® Home (measurement 1) was obtained by one of three eligible training personnel. Following a training session, the participants measured their IOP with the iCare® Home tonometer to obtain three valid measurements (measurement 2-4). The iCare® Home measurements were blinded to both the trainer and the participant, as no values are displayed on the device. Afterwards, the training personnel measured the participants’ IOP once with GAT (measurement 5). On a routine basis, 4–10 personnel (physicians, ophthalmological nurses or optometrists) worked at the clinic. In the present the study, one physician, 2 optometrists and 4 nurses participated. Within 15 min from the first GAT measurement, one of the available personnel, who was blinded to the previous results, was randomly asked to measure the IOP once with a different GAT instrument (measurement 6). All IOP measurements were made in this sequence. Finally, the iCare® Home results were downloaded by connecting the tonometer to the iCare® Link software.

One eye was randomly selected for each participant, by alternating between right eye for patient 001, left eye for patient 002 and so on. If only one eye was available, it was eligible without randomization. At least two iCare® Home measurements obtained by the participants were needed. Participants who could not perform self-tonometry due to reduced hand and arm mobility (e.g. due to rheumatism or tremor) were excluded. According to the manufacturer's recommendations, participants were excluded if the difference between the trainer's GAT measurement and the participants’ first measurement was greater than 5 mmHg and/or the range of the participants’ measurements was greater than 7 mmHg (the difference between the highest and lowest values).

### Data analysis

To detect a statistically significant difference between the methods, a sample of 41 participants was necessary (α = 0.05, β = 0.80 and S.D. 2.0 mmHg). Sample size was calculated with the G*Power program (version 3.1.9.2, University Dusseldorf, Germany). Windows Excel and SPSS version 23 (SPSS Inc., Chicago, IL, USA) were used for statistical analysis.

The data consisted of the mean and standard deviation of the IOP measured with iCare® Home (the mean of three measurements by the participants and a single measurement by the trainer) as well as with GAT (single measurements by the trainer and other personnel). Agreement between the users with each method (GAT and iCare® Home) was assessed by means of the Bland–Altman analysis.^20^ The repeatability of the measurements was assessed with a one-way analysis of variance (ANOVA). Repeatability coefficient was calculated as 2.77 × (SD (examiner 1 – examiner 2)/√2). Reliability (agreement and correlation) between measurements was assessed with the interclass correlation coefficient (ICC) based on a one-way random-effects, absolute agreement, multiple raters/measurement model.^21^ The ICC agreement scale was: < 0.50: poor reliability, 0.50–0.75: moderate reliability, 0.75–0.90: good reliability and >0.90: excellent reliability.

## Results

Sixty-one participants were recruited to the study. Seven were excluded, with five due to a difference between trainer and participant over 5 mmHg and two due to an iCare® Home range over 7 mmHg at the same session. No patient had problem handling the device due to reduced hand and arm mobility. Thirty-two female and 22 male were included with a mean age of 56 ± 17 years. Twenty-four participants were glaucoma patients and 30 were volunteers. Seven participants had tested iCare® Home once a few years previously. A total of 54 eyes from 54 participants (88%) were available for analysis.

**Figure 1.**
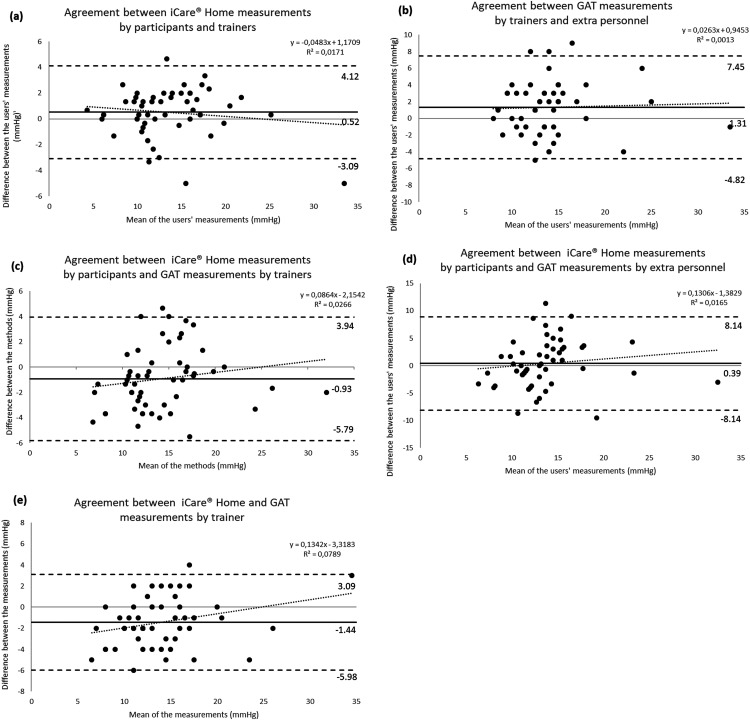
Bland Altman plots of agreement between IOP measured by different users and different methods. *Solid* lines indicate mean differences (bias); *dashed* lines indicate the upper and lower 95% limits of agreement; and *dotted* lines indicate regressions between the mean and the difference. (a) Agreement between iCare® Home measurements made by participants and trainers. (b) Agreement between GAT measurements made by trainers and extra personnel. (c) Agreement between iCare® Home measurements made by participants and GAT measurements made by trainers. (d) Agreement between iCare® Home measurements made by participants and GAT measurements made by extra personnel. (e) Agreement between iCare® Home and GAT measurements made by trainers.

The repeatability of the three consecutive measurements made by the participants using iCare® Home was excellent (ICC = 0.975, 95% CI 0.960–0.985, *p* = 0.001). Of all the measurements made by the participants using iCare® Home, 72% were within 3 mmHg to the GAT measurements made by the trainer. The Bland-Altman plot (Figure 1 a) illustrates the agreement between the methods.

The repeatability of the measurements with both methods and different users is summarized in Table 1. There was a statistically significant difference between the trainers’ measurements made with iCare® Home and those made with GAT (*p* < 0.001), as well as between the GAT measurements made by the trainers and those made by extra personnel (*p* = 0.017). The coefficients of repeatability were 2.38 for iCare® Home and 3.88 for GAT.

**Table 1.** Pairwise comparisons with one-way repeated-measure analysis of variance (ANOVA).

Methods	Mean difference	Std. error	Sig.*
iCare® Home participant vs. trainer	0.519	0.255	0.280
GAT trainer vs. extra personnel	1.315	0.421	0.017
iCare® Home participant vs. GAT trainer	−0.926	0.339	0.052
iCare® Home trainer vs. GAT trainer	−1.144	0.311	0.000
iCare® Home participant vs. GAT extra personnel	0.389	0.587	1.000

*Adjustment for multiple comparisons: Bonferroni.

Bland–Altman plots illustrating the agreement between different users for each method are shown in Figure 1b-e. The mean difference between the methods was larger with GAT than with iCare® Home, at 2.69 mmHg versus 1.48 mmHg, as was the width of the 95% limits of agreement (LoA), which ranged from −1.27 to 6.64 with GAT in comparison with −0.95 to 3.90 with iCare® Home. A regression analysis showed that the difference between the user groups became progressively greater when the IOP was above 15 mmHg. Similar results were found for both methods (iCare® Home and GAT).

The degree of reliability between the measurements is summarized in Table 2. The inter-user agreement was excellent for iCare® Home users (0.930). The inter-user agreement was moderate for GAT users (0.741).

**Table 2.** Agreement between inter-users’ measurements of IOP with iCare® Home and GAT.

	IOP mean (SD) in mmHg	ICC (95% CI)	*p*	Mean Difference*	*p*	Limits of agreement **
Home participant	14 (5)	0.930 (0.880–0.959)	0.001	1.48	0.045	−0.95–3.90
Home trainer	13 (5)					
GAT trainer	15 (5)	0.741 (0.558–0.849)	0.001	2.69	0.003	−1.27–6.64
GAT personnel	13 (5)					
Home participant vs. GAT trainer		0.852 (0.742–0.915)	0.001	2.17	0.008	−0.70–5.04
Home participant vs. GAT personnel		0.593 (0.388–0.742)	0.001	3.36	0.509	−1.93–8.65
Home trainer vs. GAT trainer		0.892 (0.821–0.936)	0.001	2.22	0.001	−0.80–5.24

*Mean absolute difference.

** Limits of agreement (LoA): Mean difference plus or minus 2 standard deviation. LoA indicates that 95% of the differences fall between these two limits.

## Discussion

The first aim of this study was to compare the inter-observer variation between GAT and iCare® Home. We found a statistically significant difference between the IOP measured with GAT by the healthcare personnel (trainer and extra personnel) and the measurements with iCare® Home by the study participants and trainers. The ICC was lower with GAT by healthcare personnel than with iCare® Home by study participants and trainers.

As opposed to most studies where GAT examiners were certified prior to the collection of the measurements, our study adopted a different approach. In order to reflect working conditions at a tertiary eye centre, all measurements were performed in routine clinical settings where none of the personal was selected and certified for the study purpose.

Despite the fact that most of the participants were first-time iCare® Home users and had a short training period, a large majority (88%) were able to perform self-tonometry with iCare Home® and could obtain valid measurements; these results were similar to those of Cvenkel et al. (82%) and better than those of Dabasia et al. (74%) and Pronin et al. (73%).^2,11,22^ The present study shows that 72% of the measurements with iCare Home® made by the participants were within 3 mmHg of the GAT measurements made by the trainer. This finding is in accordance with previous studies with glaucoma patients (78%) and healthy volunteers (68%–73%), and with a study by Huang et al. (71.5%).^1,23,24^ However, our results are slightly lower than those of Cvenkel et al. and Dabasia et al. (83.3% and 84%, respectively).^11,22^ In the latter studies, the majority of the participants were glaucoma or OHT patients, and thus were probably more familiar with IOP measurements. Our sample included a majority of healthy participants, which could partially explain the difference.

In the present study, iCare Home® tonometers showed a better repeatability than GAT and this might be caused by several factors, mainly due to the way mean IOP was calculated. On the one hand, participants’ mean IOP with iCare Home® was calculated from three measurements which are already the mean of four measurements each. The mean of these multiple measurements probably gives less variation. For the trainer, one measurement (mean of 4 measurements) was recorded. In our study, the mean difference in the inter- user measurements made with iCare Home® was 1.48 mmHg, higher than Tagaki et al. (0.21 mmHg) and Valero et al. (0.70 mmHg) where the mean was calculated from 3 measurements for both the participants and the trainer/examiner.^25,26^ On the other hand, the mean difference in the inter-user measurements made with GAT was calculated from single measurements and resulted in 2.69 ± 1.98 mmHg – a greater variation than other studies. Dielemans et al. and Tonnu et al. obtained comparable results (1.79 ± 2.41 mmHg and 1.7 ± 2.6 mmHg) despite different approaches.^26,27^ Similar to our study, Dielemans et al. compared the first measurements of two examiners, with different GAT instruments in different rooms whereas Tonnu et al. compared the means of three measurements of two examiners. Dielemans et al. and Tonnu et al. had only two examiners, trained and certified for their study. In contrast, our study reflects daily clinical practice in a tertiary eye centre where the participating personnel (three trainers and eight different personnel) was randomly selected and not study certified. This may explain the larger variations of GAT measurements compared to iCare Home®, also reflected in the repeatability coefficients (3.88 and 2.38 respectively). Interestingly, a study by Mihailovic et al. concluded that despite education and training to improve agreement between physicians’ and technicians’ IOP measurements, differences remain, indicating that GAT single measurements might be regarded with caution.^27^

Another factor that might affect repeatability is the calibration of the instruments. iCare® Home devices were calibrated at the beginning of each measurement session when GAT instruments were calibrated once a month at our clinic. Furthermore, in our study, the same iCare® Home device was used by the participant and the trainer whereas two different GAT instruments were used on the same occasion by two different healthcare staff, possibly adding to the variation of the measurements.

As shown in previous publications, a systematic bias was found between the instruments. The difference between the iCare® Home and GAT measurements made by the trainers was 2.2 mmHg, compared to the findings of Pronin et al. (2.66 mmHg), Huang et al. (–1.7 mmHg) and Dabasia et al. (1.2 mmHg).^2,22,24^ The difference between the participants’ iCare Home® measurements and the trainers’ GAT measurements was 2.17 mmHg (CI 95% −0.70–5.04), which is in accordance with the results of Pronin et al. (2.58 mmHg, CI 95% −4.71–9.88), but higher than those of Cvenkel et al. (1.2 mmHg, CI 95% −3.4–5.9) and Valero et al. (1.314 mmHg).^2,11,26^ This difference could be due to the different samples (glaucoma patients vs. healthy participants) and the design of the studies.

The level of reliability with iCare® Home measurements was excellent, with an agreement between the participants and trainers of 0.930 (CI 95% 0.880–0.959); this result was similar to the result obtained by Pronin et al. of 0.903 (CI 95% 0.867–0.928) and that obtained by Termühlen et al. of 0.891.^2,5^ The level of reliability with the GAT measurements was good, with an agreement between the trainers and personnel of 0.741 (CI 95% 0.558–0.849), in accordance with the result obtained by Dielemans et al. (0.81) but much lower than that obtained by Salim et al. (0.97) where two examiners measured three times the IOP with the same instrument.^28,29^

Our results showed a statistically significant difference between the GAT measurements made by the trainers and those made by the other personnel on the same occasion (1.315 mmHg, *p* = 0.017). This raises the question of the variation of IOP measurements with GAT at follow-up visits, when the measurements are made by different personnel. Indeed, Kawai et al. found a statistically significant difference between the variability of measurements at follow-up visits made by multiple clinicians and a single clinician (12.0% and 10.1%, *p* = 0.044).^19^

Overall, the analysis of the repeatability, agreement and reliability of iCare® Home and GAT supports the feasibility of self-tonometry. However, our study presents several limitations. First, the study was not randomized. All consecutive patients who agreed to participate in the study were included, which might induce a selection bias. Second, we did not consider parameters that can influence IOP measurements, such as corneal curvature, central corneal thickness (CCT) or corneal hysteresis (CH). Previous studies have shown a correlation between IOP measurements and CCT or CH.^1,2,22^ However, a study by Brown et al. showed similar correlations between the IOP measured by GAT and iCare® Home and CCT and CH.^3^ Our study aimed to compare the differences between inter-users within each method and CCT and CH would affect these results equally, irrespective of the users; therefore, the influence of these factors was not further studied.

Finally, three different personnel trained the participants, so the latter might have received slightly different instructions that could influence the quality of their measurements. Furthermore, the measurements were not made randomly, but were always performed in the same order. As GAT measurement by extra personnel was always measured last and delayed by up to 15 min, we cannot exclude the possibility that the IOP differed because the participants moved to another room or were anxious about meeting different personnel. We also know that repeated measurements with GAT tend to decrease the IOP.^17,28,30^ However, in our study, only two measurements were made with GAT for each participant, so this effect may be negligible.

In conclusion, our study found that tonometry with iCare® Home measured by patients has similar or less inter-user variation compared with GAT measured by healthcare professionals. IOP phasings obtained by self-tonometry with iCare® Home could be considered applicable, reliable and of great value for glaucoma management.
